# Wireless Home Blood Pressure Monitoring With Health Coaching for Hypertension Management at a Federally Qualified Health Center: Pilot Randomized Controlled Feasibility and Acceptability Study

**DOI:** 10.2196/100115

**Published:** 2026-10-22

**Authors:** Nathan Magalit, Ryan Moran, Anna Narezkina, Kevin Patrick, Anthony DeMaria

**Affiliations:** 1 Department of Medicine, Division of Cardiovascular Medicine, University of California San Diego, San Diego, CA, United States, 1 81896772759

**Keywords:** hypertension, blood pressure monitoring, telemedicine, remote patient monitoring, mobile health, Hispanic or Latino, medically underserved area, community health centers, pilot projects, feasibility studies, ambulatory

## Abstract

**Background:**

Only approximately half of adults with hypertension, a leading cardiovascular risk factor, achieve blood pressure (BP) control, with persistent disparities among low-income and minority populations. Wireless home BP monitoring (HBPM) with remote patient monitoring can improve BP control, but evidence is limited in medically underserved, predominantly Hispanic populations served by federally qualified health centers (FQHCs).

**Objective:**

The primary objective was to evaluate the feasibility and acceptability of wireless HBPM among predominantly Hispanic adults with hypertension receiving FQHC care. Comparing BP outcomes between wireless and conventional HBPM was a secondary exploratory objective.

**Methods:**

We conducted a 6-month, 2-arm randomized controlled pilot feasibility and acceptability trial nested within the San Diego: Heart Attack and Stroke Free Zone program. Adults aged ≥18 years with uncontrolled or newly diagnosed hypertension (BP ≥140/90 mm Hg) were enrolled across 3 FQHC networks and randomized 1:1 by unrestricted simple randomization to wireless HBPM (Bluetooth cuff and cellular hub; weekly transmitted summaries reviewed by health coaches) or standard HBPM (home monitor with paper log). Both arms received standardized health coaching. The primary outcomes were feasibility (proportion of 210 expected BP readings transmitted) and acceptability (postintervention satisfaction survey in the wireless arm). Exploratory analyses compared BP outcomes between arms among participants with >2 BP measurements.

**Results:**

Of 316 recruited participants, 290 (92%) were eligible, and 200 (63%) were analyzed (wireless: n=73, 23%; and standard: n=127, 40%); arm-level allocation counts were not retained. The mean age was 61 years (SD 9.1), 57% (115/200) were women, and 51% (n=102) self-identified as Hispanic, with no significant baseline differences between arms. Among analyzed wireless-arm participants, 86% (63/73) transmitted multiple readings (mean 160.6, SD 129.1) over 6 months, corresponding to 76.2% (160/210) of expected readings among transmitting participants and 66% across all 73; only 5% (7/127) of standard-arm participants returned paper logs. Of 60 respondents (82.2% response rate), 95% (n=57) agreed or strongly agreed that the device was easy to set up and use. Both arms showed significant within-arm BP reductions at 6 months (*P*<.001). At 6 months, 63% (46/73) of the wireless arm and 74% (94/127) of the standard arm achieved systolic BP <140 mm Hg (*P*=.08; adjusted odds ratio, standard vs wireless, 1.6, 95% CI 0.78‐3.4; *P*=.19). These exploratory comparisons were not powered and do not constitute evidence of equivalence.

**Conclusions:**

Among analyzed participants, wireless HBPM was feasible, and survey respondents reported high acceptability in a medically underserved, predominantly Hispanic FQHC population. Feasibility among all randomized participants could not be estimated because arm-level allocation counts were not retained. The absence of a between-arm difference in BP control is not evidence of equivalence because this pilot was not powered to compare clinical effectiveness; the BP findings are hypothesis generating.

## Introduction

Hypertension remains a significant risk factor for cardiovascular disease morbidity and mortality in the United States [1,2]. Despite numerous treatment options, epidemiologic studies show that only approximately 50% of individuals with hypertension achieve blood pressure (BP) control [2]. Disparities in who achieves BP control persist, particularly among low-income, rural, and racial or ethnic minority populations [2].

The adoption of home BP monitoring (HBPM) in clinical practice has dramatically increased over the last 20 years [3]. HBPM enables patients to measure their own BP conveniently, at frequencies and times that align with their daily routines and at relatively low cost [3]. By allowing multiple daily readings over extended periods, HBPM can help distinguish between primary hypertension, white-coat hypertension, and masked hypertension [4]. Longitudinal studies suggest that HBPM readings are stronger predictors of cardiovascular morbidity and mortality than traditional clinic BP measurements [5-8].

HBPM use has been associated with improved hypertension management outcomes [9-12]. When used to titrate hypertension medications, HBPM results in significant reductions in both systolic and diastolic BP as well as decreased overall hypertension medication use, compared to usual care [9-12]. HBPM promotes patient education on hypertension management, providing immediate feedback on management efficacy that can inform patient-directed care strategies [11].

Despite the benefits of HBPM, self-administered BP measurement with concomitant self-logging has inherent limitations, such as errors in measurement, inaccurate logging, and reporting bias, as well as unsupervised self-adjustment of prescribed medical regimens [3]. While automatic HBPM devices may address some limitations associated with traditional clinic measurement (eg, interuser variability with traditional stethoscope and sphygmomanometer), there is minimal literature on patient adherence to recommended self-measurement practices at home [3,13]. Additionally, BP data integration into electronic medical records can be challenging. Handwritten records from automatic monitors may be difficult for health care providers to interpret, susceptible to falsification, or unavailable during clinic visits if patients forget to bring them—ultimately limiting their usefulness [3].

Remote patient monitoring (RPM) uses digital devices to transmit patient health data from home, enabling patients and providers to proactively manage conditions. A key aspect of RPM involves wireless health devices capable of measuring and transmitting clinical parameters, such as BP and glucose. Specifically, wireless HBPM devices have been associated with quicker normalization of BPs compared to traditional clinic-based monitoring, better BP control compared to usual care, and potentially fewer side effects from hypertension medication adjustment [14,15]. Importantly, studies have shown that patient acceptance of wireless health devices tends to be high [14,15]. However, significant sociodemographic disparities exist in the adoption of digital health technologies, with lower uptake observed among racial or ethnic minorities, older adults, and individuals from lower socioeconomic backgrounds [16]. These disparities highlight the importance of targeted initiatives to promote equitable access and adoption of wireless HBPM, particularly within underserved and high-risk communities that could derive the greatest benefit from improved BP management.

Little information is available on using wireless HBPM in community-based interventions with medically underserved, high-risk populations. These data are even more limited among the Hispanic population. To address this gap in knowledge, we conducted an RPM pilot study nested within a larger quality improvement program (“San Diego: Heart Attack and Stroke Free Zone”) that was designed to increase adherence to guidelines for cardiovascular risk factor modification and improve cardiovascular outcomes among underserved populations [17]. The primary objective of this pilot was to determine whether wireless HBPM could be feasibly deployed in, and would be acceptable to, an underserved, predominantly Hispanic federally qualified health center (FQHC) population. Feasibility was operationalized as the proportion of expected BP readings successfully transmitted, and acceptability was operationalized as participant-reported experience with the device. As a secondary, exploratory objective, we described BP change and BP control in each arm. The study was not designed to test the clinical effectiveness of wireless HBPM relative to conventional HBPM, and no formal hypothesis of superiority or equivalence was tested.

## Methods

### Study Design and Enrollment

We conducted a 6-month prospective, 2-arm randomized controlled pilot feasibility and acceptability trial within the “San Diego: Heart Attack and Stroke Free Zone” (HSF-Z) program [17]. HSF-Z was a Center for Medicare and Medicaid Innovation–funded multicenter study aimed at developing and evaluating a comprehensive cardiovascular risk reduction program, consisting of intensive health coaching and a predefined regimen of medical therapy to decrease the incidence of myocardial infarction and cerebrovascular disease throughout greater San Diego County. This cardiovascular risk reduction program included participants if they (1) were aged ≥50 years with a BP of >140 mm Hg systolic or 90 mm Hg diastolic; (2) were aged ≥50 years with diabetes; (3) had a 10-year atherosclerotic cardiovascular disease risk score of ≥7.5%; and (4) had any cardiovascular disease at any age. Patients within the HSF-Z study population were eligible for our substudy if they were aged ≥18 years and had BP of 140 mm Hg systolic or 90 mm Hg diastolic measured by 3 consecutive measurements within at least 5 minutes of each other. Participants were eligible if they had known hypertension but were not at goal on their current regimen, or if they had a new diagnosis of hypertension. The trial was conducted as a nested pilot substudy of the HSF-Z quality improvement program and was not prospectively registered in a public clinical trials registry. Reporting follows the CONSORT-EHEALTH (Consolidated Standards of Reporting Trials of Electronic and Mobile Health Applications and Online Telehealth) checklist (Checklist 1) [18].

Participants were enrolled from clinics in 3 FQHC networks across San Diego County. Health centers were selected to include a high proportion of Hispanic patients. These clinics predominantly see underserved individuals with Medicaid (Medi-Cal) health insurance. Recruitment, consent, training, and outcome assessment were all conducted in person at the clinic sites.

If identified as eligible, individuals were invited to participate; if interested, participants were randomized at each study site to either arm based on an a priori created 1:1 allocation list. The allocation list was generated before the start of enrollment by the study team using a spreadsheet random number function to assign the value 0 or 1 across sequential cells, corresponding to an intended 1:1 allocation ratio. This constituted unrestricted simple randomization: no blocking, stratification, or minimization was applied. A single allocation sequence, held in a shared spreadsheet, was used across all 3 participating FQHC networks, with clinic-based study staff at each site assigning the next unused entry as participants enrolled. Whether upcoming assignments were concealed from enrolling staff before each participant was enrolled cannot be confirmed from available study records.

The intervention required dispensing physically distinct monitoring equipment; participants and site staff could not be blinded after allocation, and health coaches were also unblinded, as delivery of weekly aggregated wireless BP summaries to the clinical site was itself a component of the intervention. Outcome ascertainment relied on clinic BP measurements obtained as part of routine HSF-Z program care rather than on assessments made specifically for this substudy.

### Sample Size

No formal power calculation was performed, and no target enrollment was set. Consistent with the pilot and feasibility objectives, the study aimed to recruit as many eligible participants as the participating clinics could accommodate, and enrollment was limited in practice by clinic recruitment capacity and available research site resources. Recruitment yielded 316 participants over the 2-year enrollment period. The study was not sized to detect between-arm differences in BP or BP control, and no interim or futility analyses were planned or performed.

### Interventions

#### Wireless Arm

Participants in the wireless intervention received a 30- to 60-minute training on the appropriate use of the HBPM system (2NET system; Qualcomm Life; and UA-651 BLE monitor; A&D Engineering, Inc). This system included a BP cuff with wireless transmission capabilities, which automatically synced via Bluetooth with the secure 2NET hub, and BP readings were uploaded to a protected server. Data were aggregated in weekly summaries and provided to the clinical site for health coach review. Participants in the wireless arm were instructed that although data were collected each time they used their cuff, they were only evaluated by their provider on a weekly basis. There were no prompts or reminders provided by the BP monitoring system to encourage data collection.

#### Standard Arm

The standard BP cohort received a standardized BP log and home BP monitor (A&D UA-767PBT or comparable model or device), which was appropriately calibrated at the time of enrollment. Participants were trained on the appropriate use of a home BP monitor by clinical staff (medical assistant, licensed vocational nurse, or registered nurse) and instructed to log their BP daily at home and bring the log to their clinic visits.

#### Health Coaching (Provided to Both Arms)

Health coaches involved in the larger HSF-Z cardiovascular risk reduction study did not alter their approach to participants in this pilot study, with the exception that the wireless data were collected and aggregated weekly and provided to the clinic site for review. Health coaches followed a standardized protocol (Multimedia Appendix 1), including weekly check-ins via telephone to inquire about (1) medication adherence and pill counts to ensure appropriate medication supply and (2) BP follow-up. Once a patient was determined to be adherent to their medication regimen and BP was controlled, health coach encounters switched to monthly. Coaching dose was therefore identical by protocol in the 2 arms: weekly telephone contacts during the active titration phase, de-escalating to monthly contacts once medication adherence and BP control were established, for the full 6-month period. Coaching content, contact frequency, and escalation criteria did not differ by arm; the only protocolized difference between arms was that weekly aggregated wireless BP summaries were made available to the clinical site for wireless-arm participants. Coaching was delivered by FQHC-based staff in the participant’s preferred language, including Spanish. Health coaches were not blinded to study arm because receipt of transmitted BP summaries was intrinsic to the intervention.

### Measures

Baseline characteristics were collected at enrollment, including demographics (age, years of education, employment, and race or ethnicity) and comorbidity burden (tobacco use, diabetes, and cholesterol levels) as part of the overarching HSF-Z study on cardiovascular risk reduction. The target for BP was defined based on the Eighth Joint National Committee (JNC8) guidelines (the most current at the time of the study design) as <140/90 mm Hg for participants aged <60 years and <150/90 mm Hg for those aged ≥60 years [19]. These thresholds were also consistent with the Healthcare Effectiveness Data and Information Set (HEDIS) quality measures in effect when the study was designed, which were the benchmarks against which the participating FQHCs were evaluated; using them therefore aligned the study target with the care standard participants were actually receiving [20]. For descriptive reporting of BP control in the exploratory analyses, we additionally applied a single uniform threshold of systolic BP <140 mm Hg to all participants regardless of age. This is the systolic component of the stricter of the 2 JNC8 age-specific targets and was chosen to provide one conservative, unambiguous summary measure across a cohort in which the mean age (61, SD 9.1 years) fell close to the JNC8 age cut point.

At enrollment, BPs were measured clinically and again at 3 months and 6 months (end of the pilot). Measures were collected by study sites and uploaded to a centralized, Health Insurance Portability and Accountability Act (HIPAA)–compliant data server. All participants were instructed at the time of enrollment to measure their BPs twice daily for the first month and then daily at home. This measurement cadence was chosen to balance data quality with participant burden, maximizing adherence while preserving clinical value. In day-to-day practice, a single daily reading gives clinicians enough lead time to detect and address out-of-range values within 24 hours. Requiring twice-daily readings can enrich study data, but the additional workload often reduces long-term adherence and increases behavioral fatigue. Regardless of grouping, participants were told to contact the clinic if their measurements met any of the following criteria: systolic BP of <100 mm Hg or >170 mm Hg, diastolic BP of <50 mm Hg and/or >100 mm Hg or if they were symptomatic.

### Outcomes

One primary outcome was feasibility, defined as the proportion of expected BP readings successfully transmitted by participants in the wireless arm over the 6-month study period. Expected readings were derived directly from the prescribed measurement schedule: 2 readings per day for the first 30 days (60 readings) plus 1 reading per day for the remaining approximately 150 days (150 readings), for a total of 210 expected readings per participant. Readings were counted as received if they were transmitted by the cuff, relayed by the 2NET hub, and recorded on the study server.

The other primary outcome was acceptability, assessed in the wireless arm at the end of the pilot using an investigator-developed satisfaction survey. Participants rated their agreement with statements addressing ease of equipment setup, ease of cuff use, ease of viewing one’s own readings, and anticipated preference for the wireless versus the conventional cuff.

Exploratory outcomes were mean clinic systolic and diastolic BP, change in clinic BP from baseline at 3 and 6 months, and the proportion of participants achieving systolic BP <140 mm Hg at 6 months. These outcomes were analyzed descriptively and compared on an exploratory basis, not to establish effectiveness.

### Participant Enrollment

In total, 316 individuals were identified as interested in enrollment and recruited between February 2015 and February 2017, of whom 26 did not satisfy enrollment criteria, leaving 290 who met eligibility criteria. Eligible individuals were randomized; however, the total number randomized and the number allocated to each arm were not retained in the summary data available to the investigators. After allocation, 48 participants were withdrawn, usually because of inability to contact the participant despite numerous efforts, and 42 lacked at least two clinically collected BP measurements (ie, missing either the 3-month or the 6-month clinic measure). The remaining 200 participants constituted the analyzed sample: 73 in the wireless arm and 127 in the conventional arm.

Accordingly, 73 and 127 are the numbers of analyzed participants in each arm, not the numbers randomized. Arm-level counts of withdrawal and of exclusion due to insufficient clinic BP measurements were not retained in the summary dataset available to the investigators and are therefore reported in aggregate; missing follow-up BP measurements within the analyzed sample are reported by arm and time point in the Results.

### Study Disenrollment

At 6 months, all participants were disenrolled from this pilot study but continued their routine follow-up as part of the larger HSF-Z trial; therefore, their 12-month follow-up BP data were available. Those in the standard treatment group were allowed to keep their home cuffs, and those randomized to the wireless arm were provided with an equivalent conventional cuff upon return of the wireless cuff and 2NET hub. Participants in the wireless arm completed a survey regarding their experience with using the wireless HBPM.

### Statistical Analysis

The primary feasibility and acceptability outcomes were summarized descriptively in the wireless arm without between-arm comparison; inferential testing was applied only to the exploratory BP analyses.

Continuous variables are expressed as mean (SD), whereas categorical variables are presented as absolute counts and percentages. Differences between categorical variables were analyzed using the chi-square or Fisher exact test, as appropriate, and differences between continuous variables using the Student *t* test.

Clinically collected BPs were used to determine changes between the study arms, and only participants with 2 or more clinic visits were included in the analysis. For the exploratory analysis of BP control, the dependent variable was achievement of systolic BP <140 mm Hg at the 6-month visit, modeled by logistic regression among participants with a 6-month clinic measurement (wireless: 66/73, 90%; standard: 101/127, 80%). Study arm was the independent variable of interest, with the wireless arm as the reference category, so that the adjusted odds ratio (AOR) expresses the odds of BP control in the standard arm relative to the wireless arm. Candidate covariates were the baseline characteristics listed in Table 1; those found to have a statistical association with BP control at 6 months were retained in the final model. The specific covariates retained, event counts per covariate, handling of missing covariate values, and model diagnostics were not retained in the summary output available to the investigators and cannot be reported. Analyses were complete case, restricted to participants with the relevant measurement available, rather than intention-to-treat analyses of all randomized participants. Net changes from baseline are reported among participants with both a baseline and the corresponding follow-up measurement; because the number of participants with follow-up measurements differed by visit, paired change estimates do not equal the difference between the arm-level means reported at each visit.

**Table 1.** Baseline demographic and clinical characteristics of analyzed participants (n=200) by study arm in a 6-month pilot randomized controlled feasibility and acceptability trial of wireless versus conventional home blood pressure monitoring among adults with uncontrolled hypertension at 3 federally qualified health center networks in San Diego County, California (2015‐2017)^a^.

Baseline characteristic	Wireless analyzed (n=73)	Standard analyzed (n=127)	*P* value^b^
Age (y), mean (SD)	60.1 (9.5)	61.3 (8.9)	.38
Gender (women), n (%)	40 (54.8)	75 (59.1)	.66
Ethnicity (Hispanic), n (%)	39 (53.4)	63 (49.6)	.66
Race, n (%)			.44
White	63 (90)	113 (91.9)	
African American	6 (8.6)	5 (4.1)	
Pacific Islander	0 (0)	1 (0.8)	
Asian	1 (1.4)	4 (3.3)	
Income, n (%)			.16
<$10,000	11 (57.9)	18 (32.7)	
$10,000‐$30,000	8 (42.1)	35 (63.6)	
>$100,000	0 (0)	2 (3.6)	
Spanish-speaking, n (%)	32 (45.1)	50 (39.4)	.46
Marital status (married), n (%)	19 (57.6)	36 (48.6)	.41
Employment (unemployed), n (%)	22 (66.7)	61 (80.3)	.15
Smoking, n (%)	19 (26.4)	22 (17.3)	.15
Diabetes mellitus, n (%)	34 (47.2)	71 (55.9)	.30
History of CAD^c^, n (%)	2 (2.7)	4 (3.2)	>.99
History of MI^d^, n (%)	6 (8.8)	5 (4)	.20
History of PVD^e^, n (%)	0 (0)	3 (2.4)	.30
History of CVA^f^, n (%)	2 (2.9)	14 (11.3)	.06
Baseline LDL^g^ >100, n (%)	35 (57.4)	61 (56)	.87
Baseline A_1C_ ≥8, n (%)	11 (21.2)	19 (20.9)	>.99
Baseline BMI, mean (SD)	32.2 (6.9)	31.0 (6.8)	.22
Baseline A_1c_, mean (SD)	7.0 (1.7)	7.2 (1.9)	.54
Total cholesterol, mean (SD)	189.8 (37.1)	187.0 (40.3)	.63
LDL, mean (SD)	108.4 (30.2)	102.9 (35)	.27
HDL^h^, mean (SD)	49.9 (15.2)	51.4 (15.4)	.51

a Data were incomplete for this variable; percentages are calculated using the number of participants with available data (wireless and standard arms, respectively): race, 70 and 123; reported family income, 19 and 55; preferred language, 71 and 127; marital status, 33 and 74; employment status, 33 and 76; current smoking, 72 and 127; diabetes mellitus, 72 and 127; baseline LDL >100 mg/dL, 61 and 109; and baseline HbA1c ≥8%, 52 and 91. Age, gender, and ethnicity were complete for all 200 analyzed participants.

b *P* values were calculated using the Fisher exact test for categorical variables and the 2-sided *t* test for independent means for continuous variables.

c CAD: coronary artery disease.

d MI: myocardial infarction.

e PVD: peripheral vascular disease.

f CVA: cerebrovascular accident.

g LDL: low-density lipoprotein.

h HDL: high-density lipoprotein.

A 2-tailed *P* value (*P*<.05) was considered statistically significant. All analyses were performed using SAS (version 9.4; SAS Institute Inc) and SPSS (version 21; IBM Corp).

Exploratory analyses of BP control outcomes were included but were not powered for definitive conclusions.

### Missing Data

All 200 analyzed participants had a baseline clinic BP measurement by design. Within the analyzed sample, follow-up clinic BP was missing for 8 (11%) of 73 wireless-arm participants and 10 (7.9%) of 127 standard-arm participants at 3 months, and for 7 (9.6%) of 73 and 26 (20.5%) of 127, respectively, at 6 months; overall, 9% of 3-month and 16.5% of 6-month clinic BP values were missing. Baseline covariate data were also incomplete for several socioeconomic variables collected by the parent program, most notably reported family income (available for 74/200, 37%, participants); denominators for each variable are given in Table 1.

We were unable to perform a formal test of whether these data were missing completely at random (MCAR) or to apply multiple imputation because the individual-level dataset required for either procedure was destroyed in accordance with the institutional data retention policy in effect at study completion; only aggregate summary output remains available to the investigators.

### Ethical Considerations

This study was reviewed and approved by the University of California, San Diego, Institutional Review Board (protocol 150190). The study was conducted between 2015 and 2017.

Eligible patients identified through participating FQHC clinics were invited to participate, and written informed consent was obtained from each participant prior to enrollment and randomization.

To protect participant privacy and confidentiality, all clinical and home-transmitted BP measurements were uploaded to a centralized, HIPAA-compliant data server with role-based access restricted to authorized study personnel. Wireless transmissions from the Qualcomm Life 2NET hub and A&D UA-651 BLE monitor occurred over encrypted connections, and all data used for analysis were deidentified. Individual-level study data were subsequently destroyed in accordance with the institutional data retention policy in effect at the time of study completion.

Participants received no financial compensation for participation in this pilot substudy.

## Results

### Enrollee Characteristics

The participant flow is shown in Figure 1. In total, our study included 200 analyzed participants, 73 (36%) in the wireless BP arm and 127 (64%) in the conventional BP arm (Table 1); these are analyzed rather than randomized totals. The average age of the study population was 61 (SD 9.1) years, and most (n=115, 57%) were women. More than 50% (n=102) of the study population self-identified as Hispanic (wireless arm: 39/73, 53.4% vs conventional arm: 63/127, 49.6%). There were no differences by study arm in sociodemographic parameters (eg, age, race, reported family income, gender, preferred language, marital status, employment status, and current smoking). No differences were seen in terms of reported comorbidities (diabetes, history of coronary artery disease, prior myocardial infarction, or history of stroke) or baseline laboratory values between the groups. Several baseline variables collected by the parent program were incompletely recorded; the denominator for each such variable is given in the footnote to Table 1, and percentages are calculated using those denominators rather than the full arm sizes. Reported family income was the least complete variable, available for 19 (26%) of 73 wireless-arm participants and 55 (44%) of 127 standard-arm participants.

**Figure 1.**
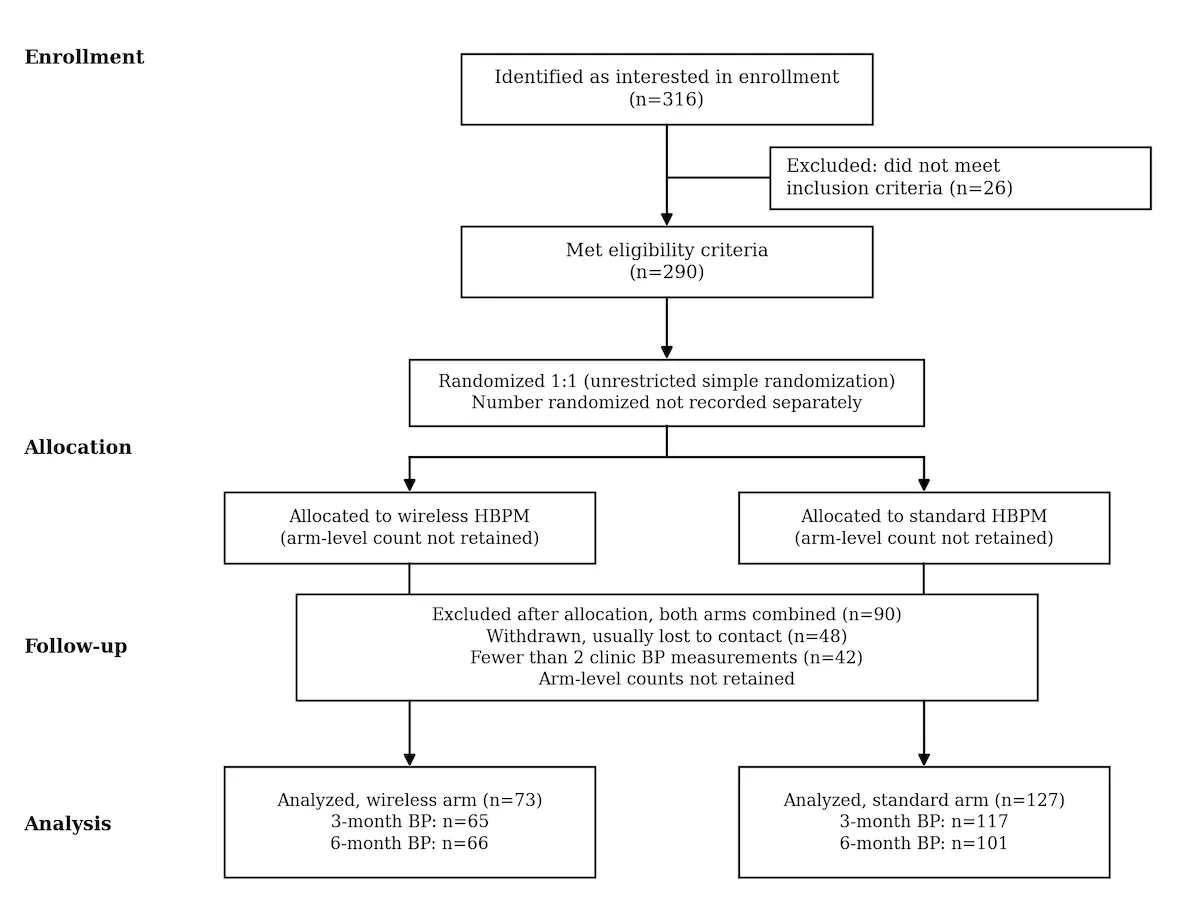
CONSORT (Consolidated Standards of Reporting Trials) flow diagram of participant recruitment, eligibility, randomization, and analysis in a 6-month pilot randomized controlled feasibility and acceptability trial of wireless versus conventional home blood pressure (BP) monitoring (HBPM) among adults with uncontrolled hypertension at 3 federally qualified health center networks in San Diego County, California (recruitment February 2015 to February 2017). The total number randomized, the number allocated to each arm, and arm-level exclusions after allocation were not retained and are reported in aggregate.

### Wireless Arm Experience

Just more than 86% (63/73) of individuals in the wireless arm were able to successfully use the device and transmit the BP readings on multiple occasions, which were collected throughout the pilot. Of these 63 participants, there was a mean of 160.6 (SD 129.1) recordings submitted throughout 6 months, which translates to an average submission adherence of 76.2% (average of 160 submissions of 210 scheduled submissions). This 76.2% figure is conditional on participants transmitting any data: its denominator is the 210 expected readings for each of the 63 participants from whom readings were received. A total of 10 participants (13.7% of the 73 analyzed wireless-arm participants) transmitted no usable data and are excluded from that estimate. Counting those participants as having contributed zero readings gives a more conservative overall transmission rate of 66% of all expected readings across the analyzed wireless arm (approximately 10,118 readings received of 15,330 expected). In comparison, 7 (5%) BP logs were collected in the conventional BP arm.

All individuals in the wireless arm were offered a satisfaction survey to inquire about ease of use and preferences. Of those who responded (60 of the 73 analyzed wireless-arm participants, 82.2%), 95% (57/60) reported “agree” or “strongly agree” with the statements “I had no problems setting up the equipment,” “I had no problems using the wireless cuff,” and “I could easily see my blood pressure readings.” Importantly, 75% (45/60) of respondents noted “agree” or “strongly agree” to “I think I would more consistently use the wireless cuff than the regular cuff.”

No harms or adverse events attributable to the intervention were observed or reported, and no privacy or security breaches were identified.

### BP Measurements and Control: Exploratory Analyses

As presented in Table 2, at enrollment, no between-group differences were seen in mean BP (systolic and diastolic) in the wireless arm versus the standard arm (159.0/86.2, SD 16.7/13.2, and 156.7/85.1, SD 15.0/11.6 mm Hg, respectively). At 3 months, similar reductions were seen in mean BPs in both groups: −22.1/−7.2 mm Hg (wireless arm) and −19.5/−8.2 mm Hg (conventional arm; *P*=.34 and *P*=.58 for systolic and diastolic BP, respectively). These similarities in reductions persisted throughout the pilot as shown in Figures 2 and 3. By the end of the pilot, 63% (46/73) of the wireless arm and 74% (94/127) of the conventional arm achieved systolic BP <140 mm Hg (among participants with a 6-month clinic measurement; wireless: 66/73, 90%; standard: 101/127, 80%), which was not statistically different (*P*=.08). Results from the adjusted multivariable model yielded no statistical differences between BP control in 2 study arms (standard vs wireless arm AOR 1.6, 95% CI 0.78‐3.4; *P*=.19). After adjustment, participants in the standard arm had 1.6 times the odds of achieving systolic BP <140 mm Hg at 6 months compared with those in the wireless arm; because the 95% CI includes 1, the data are compatible with higher odds of control in either arm.

**Table 2.** Clinic blood pressure at baseline, 3 months, and 6 months by study arm among analyzed participants in a 6-month pilot randomized controlled feasibility and acceptability trial of wireless versus conventional home blood pressure monitoring at 3 federally qualified health center networks in San Diego County, California (2015‐2017)^a^.

Blood pressure metric	Wireless arm (n=73)		Standard arm (n=127)		*P* value^b^
	n (%)	Mean (SD)	n (%)	Mean (SD)	
Mean baseline systolic pressure (mm Hg)	73 (100)	159.0 (16.7)	127 (100)	156.7 (15.0)	.30
Mean baseline diastolic pressure (mm Hg)	73 (100)	86.2 (13.2)	127 (100)	85.1 (11.6)	.54
Mean systolic pressure at 3 months (mm Hg)	65 (89)	136.4 (16.0)	117 (92)	137.7 (16.7)	.60
Mean diastolic pressure at 3 months (mm Hg)	65 (89)	79.4 (9.1)	117 (92)	77.3 (8.3)	.11
Mean systolic pressure at 6 months (mm Hg)	66 (90)	136.9 (17.3)	101 (80)	136.4 (19.4)	.87
Mean diastolic pressure at 6 months (mm Hg)	66 (90)	78.7 (12.8)	101 (80)	76.5 (9.0)	.21
Mean net systolic pressure difference at 3 months (mm Hg)^c^	65 (89)	−22.1 (18.8)	117 (92)	−19.5 (17.5)	.34
Mean net diastolic pressure difference at 3 months (mm Hg)^c^	65 (89)	−7.2 (11.3)	117 (92)	−8.2 (11.4)	.58

a Values are complete case, and the number of participants contributing to each estimate is shown in each cell. All analyzed participants had a baseline measurement; follow-up measurements were missing for some participants at 3 and 6 months (see “Missing Data” section).

b Between-arm comparisons used the 2-tailed *t* test for independent means. All comparisons are exploratory and were not powered to detect between-arm differences.

c As baseline means include all analyzed participants and follow-up means include only those measured at that visit, net changes do not equal the difference between the visit means.

**Figure 2.**
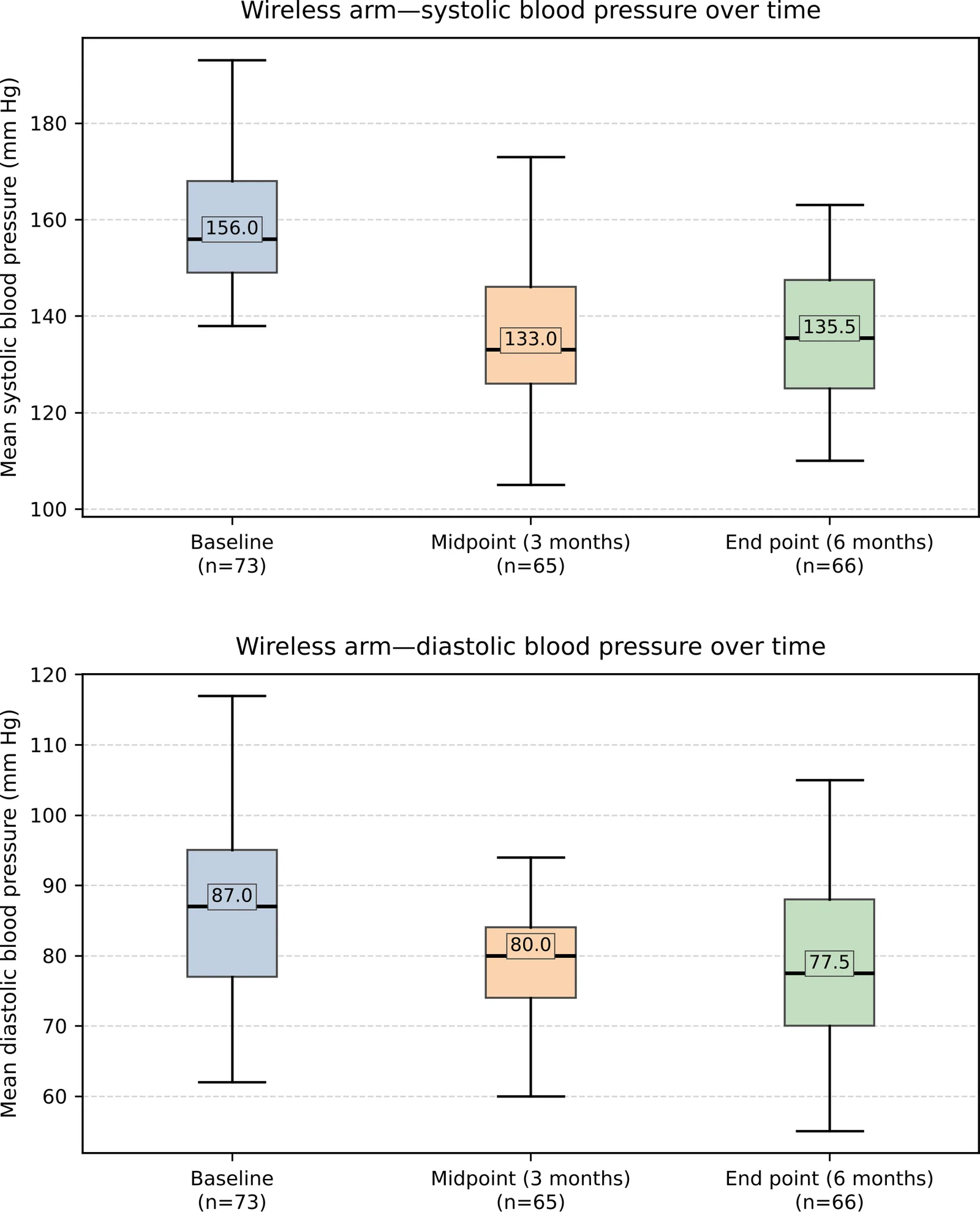
Clinic systolic (top) and diastolic (bottom) blood pressure at baseline (n=73), 3 months (n=65), and 6 months (n=66) among analyzed participants in the wireless home blood pressure monitoring arm of a 6-month pilot randomized controlled feasibility and acceptability trial among adults with uncontrolled hypertension at 3 federally qualified health center networks in San Diego County, California (2015‐2017). Boxes show the IQR; the horizontal line and numeral indicate the median. Box fill color denotes time point: blue, baseline; orange, 3 months; and green, 6 months.

**Figure 3.**
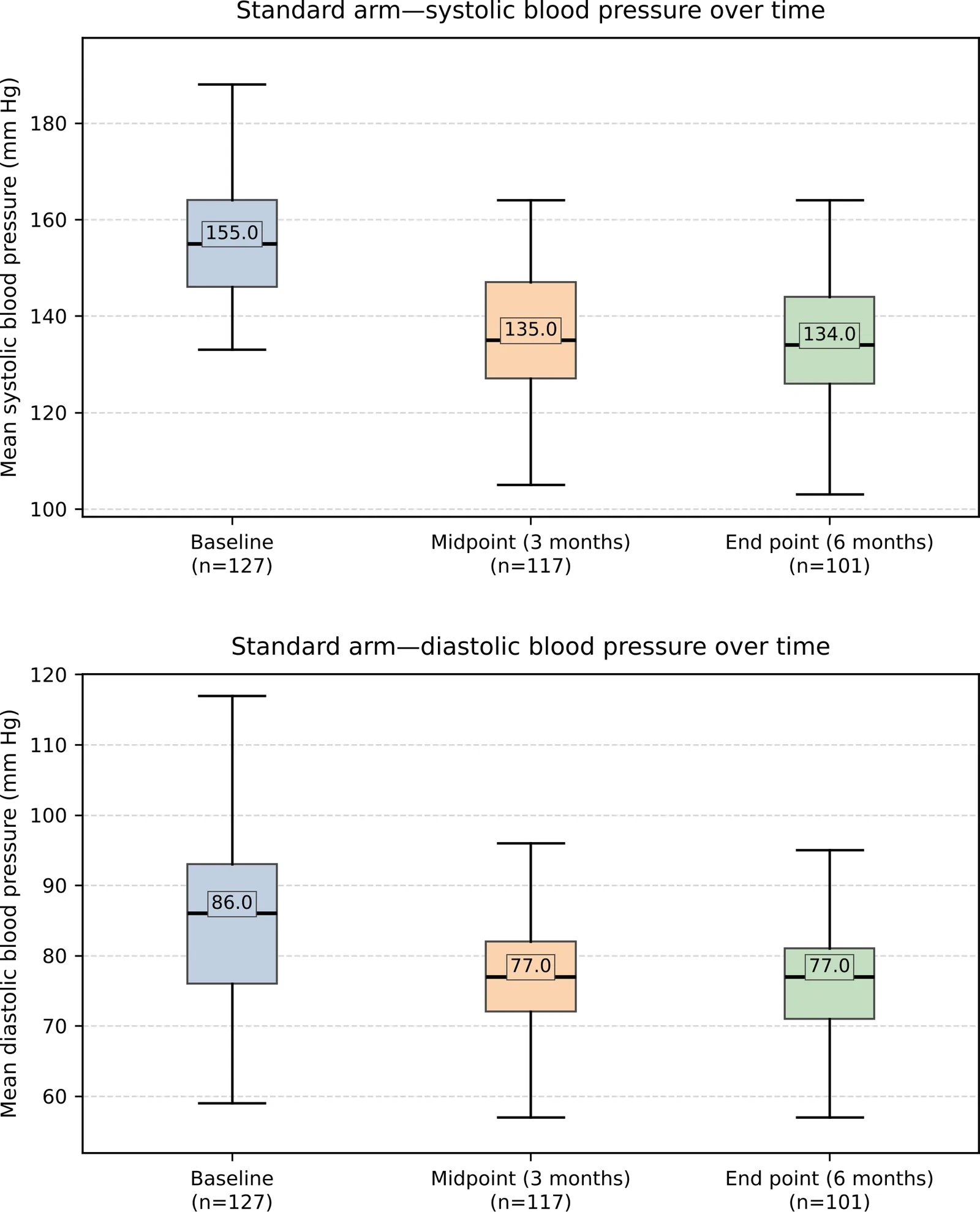
Clinic systolic (top) and diastolic (bottom) blood pressure at baseline (n=127), 3 months (n=117), and 6 months (n=101) among analyzed participants in the conventional home blood pressure monitoring arm of a 6-month pilot randomized controlled feasibility and acceptability trial among adults with uncontrolled hypertension at 3 federally qualified health center networks in San Diego County, California (2015‐2017). Boxes show the IQR; the horizontal line and numeral indicate the median. Box fill color denotes time point: blue, baseline; orange, 3 months; green, 6 months.

Mean systolic and diastolic BPs decreased significantly from baseline to 6 months in both the wireless and conventional arms. For systolic BP, the wireless arm demonstrated a mean net reduction of 15.1 mm Hg, while the conventional arm demonstrated a mean net reduction of 16.1 mm Hg (*P*<.001 for both). For diastolic BP, the wireless arm demonstrated a mean net reduction of 5.7 mm Hg, and the conventional arm demonstrated a mean net reduction of 5.9 mm Hg (*P*<.001 for both).

## Discussion

### Principal Findings

Our study evaluated a community-based, technologically driven approach to BP management, nested within an overarching cardiovascular risk reduction program. To our knowledge, this is the first such program targeting primarily an underserved population with a large Hispanic presence before the COVID-19 pandemic. With respect to the primary objectives, wireless HBPM proved deployable among analyzed participants: 86% (n=63) of the 73 analyzed wireless-arm participants transmitted readings, transmission covered 76.2% of expected readings among those participants and 66% of expected readings across all analyzed wireless-arm participants, and 95% (n=57) of survey respondents reported no difficulty setting up or using the equipment. As arm-level allocation counts were not retained, these feasibility estimates describe the analyzed sample rather than all participants originally allocated to wireless monitoring, and the acceptability estimates describe the 60 survey respondents only. With respect to the secondary, exploratory objective, both arms showed substantial within-arm BP reductions, and we observed no statistically significant between-arm difference in BP control, a result we interpret as hypothesis generating rather than as evidence about the comparative effectiveness of the 2 monitoring approaches.

### Comparison With Prior Work

We observed no significant difference between the wireless and conventional arms. This contrasts with prior studies evaluating wireless HBPM at the time of study completion, which reported better or improved control compared with the conventional approach [14,15]. In TASMINH2, conducted in the United Kingdom and primary care settings, self-monitoring with telemonitoring and patient self-titration of medication reduced systolic BP by 12.9 mm Hg at 6 months, compared with 9.2 mm Hg under usual care, a between-group difference of 3.7 mm Hg that widened to 5.4 mm Hg at 12 months [14]. In the HyperLink cluster randomized trial, home BP telemonitoring combined with pharmacist case management achieved BP control at both 6 and 12 months in 57.2% of intervention participants compared with 30% under usual care [15]. Both trials paired transmitted home readings with a defined mechanism for acting on them—patient self-titration in TASMINH2 and pharmacist-directed medication adjustment in HyperLink—and both compared that combination with usual care. Our study differed on both counts: our approach relied on passive data collection with weekly review of aggregated summaries, and the comparator arm received an HBPM and the same intensive health coaching rather than usual care. The populations also differed. HyperLink enrollees were 82% White, whereas our cohort was predominantly Hispanic, largely Medicaid insured, and recruited from safety-net clinics, a population in which telemonitoring trials have rarely been conducted.

Individual patient meta-analyses found modest BP reductions with self-monitoring and suggested that home readings help overcome therapeutic inertia, which offers the most direct explanation for the absence of a between-arm difference in our study [9,10]. Read against that evidence, both of our arms were given an HBPM, and both received intensive coaching; they differed only in how home readings were captured and transmitted. The existing evidence would predict little difference between arms structured this way, which is consistent with what we observed, and the substantial within-arm reductions in both groups are consistent with the larger effects reported when self-monitoring is combined with intensive support.

The high acceptability reported by survey respondents is consistent with a broader literature emphasizing that the impact of remote monitoring depends heavily on usability, user-centered design, and the adaptability of digital devices to patients with varying digital literacy and physical capability [21]. The system studied here required no smartphone, no app, no account creation, and no active data entry: the cuff paired automatically with a hub that transmitted readings without further user action. We suspect this minimal interaction burden contributed materially to the transmission rates and satisfaction observed in a low-income population with mixed digital literacy, and it argues for prioritizing low-interaction designs when selecting RPM platforms for FQHC settings. Conversely, the 10 wireless-arm participants who transmitted no data are a reminder that even low-burden technology does not reach everyone and that programs need a defined fallback pathway for patients who do not engage with the device.

### Interpretation and Implications

Several features of our design plausibly account for the absence of a between-arm difference. Most importantly, participants in both arms received the same structured health coaching and the same prespecified medical regimen as part of the parent HSF-Z program, including weekly telephone contacts addressing medication adherence, pill counts, and BP follow-up. Coaching of this intensity is itself an active intervention with well-documented effects on BP, and here, it was delivered to the control arm as fully as to the intervention arm. The large within-arm reductions we observed in both groups are therefore most plausibly attributable in substantial part to that cointervention rather than to the monitoring modality, and the design leaves the incremental contribution of wireless monitoring over and above coaching unresolved. A related consideration is that participation itself may have changed behavior: participants in both arms knew they were enrolled in a monitoring study, were given a home BP device, and had substantially more clinical contact than usual care would provide. Observation effects and increased clinical engagement of this kind would be expected to improve BP in both arms and to compress any difference between them. Finally, our approach was oriented toward passive data collection with weekly review rather than real-time, algorithm-driven response; contemporary RPM programs that trigger immediate clinical action on out-of-range readings may perform differently.

A further observation from this pilot is that only approximately 5% (7/127) of conventional BP logs were returned, yet BP control in the standard arm was numerically similar to—and if anything slightly higher than—that in the wireless arm. We are cautious about drawing inference from this contrast. The difference in control was not statistically significant, follow-up was less complete in the standard arm, and the 2 adherence measures capture different constructs. What the low return rate may suggest is that paper-log retrieval is an unreliable mechanism for getting home BP data in front of a clinician in this setting, independent of whether patients are measuring BPs at home. Some differences were observed among subgroups of our study population, but given the size of this pilot, we regard them as hypothesis generating and do not report them as findings.

### Limitations

This study has several important limitations. First and most fundamentally, it was a pilot trial designed to evaluate feasibility and acceptability, and it was neither designed nor powered to compare clinical effectiveness. No power calculation was performed for a BP end point, and the CI around the AOR for BP control (0.78‐3.4) is wide enough to include clinically important benefit in either direction. The absence of a statistically significant between-arm difference is therefore uninformative about effectiveness and should not be read as evidence of equivalence; equivalence or noninferiority claims would require a prospectively specified margin and a sample size well beyond that of this pilot.

Second, attrition was substantial, and its consequences cannot be fully characterized. Of 316 participants recruited, 116 (36.7%) did not contribute analyzable data: 26 did not satisfy enrollment criteria, 48 were withdrawn during the study, and 42 lacked at least two clinically collected BP measurements (ie, missing either the 3-month or the 6-month clinic measure). As the number allocated to each arm was not retained, the feasibility estimates of 76.2% and 66% are calculated among the 73 analyzed wireless-arm participants rather than all participants originally allocated to wireless monitoring; participants who withdrew or lacked follow-up clinic measurements may have transmitted fewer readings, so feasibility in the randomized population cannot be estimated reliably and may be lower than reported. We were unable to compare analyzed participants with excluded participants on baseline characteristics. This pilot was conducted as a nested cohort within the San Diego: Heart Attack and Stroke Free Zone program; the underlying clinical data were owned by the participating FQHCs and were reported to the study team only as available in the clinics and as permitted by the research protocol, and data on excluded participants were not among the data released to the investigators. The analyzed sample is therefore enriched by construction for participants who remained reachable and attended follow-up visits, and both the feasibility estimates and the exploratory BP results may be optimistic relative to what an unselected FQHC population would achieve. The direction and magnitude of this selection bias cannot be quantified with the data available, and it is the single largest constraint on the generalizability of our findings.

Third, our analyses were complete case rather than intention-to-treat, which departs from the analytic principle normally applied to randomized trials and can bias estimates when missingness is related to outcome. Missing 6-month clinic BP was approximately twice as common in the standard arm (26/127, 20.5%) as in the wireless arm (7/73, 9.6%), so the 2 arms’ 6-month estimates rest on differently selected subsets of participants. We could not test whether these data were MCAR, nor apply multiple imputation, because the individual-level dataset required for either procedure was destroyed under the institutional retention policy in force at study completion.

Fourth, allocation concealment was uncertain, and allocation was imbalanced. The allocation sequence was held in a spreadsheet accessible to enrolling clinic staff, and we cannot confirm that upcoming assignments were concealed before each participant was enrolled. If staff could view the next assignment, decisions about whom to enroll, or when, could have been influenced by knowledge of the upcoming arm, introducing selection bias of unknown direction. The analyzed sample was also imbalanced (73 vs 127). Unrestricted simple randomization without blocking or stratification does not constrain arm sizes, and differential withdrawal and follow-up completeness then compounded the imbalance. Although measured baseline characteristics did not differ significantly between arms, the smaller wireless arm carries correspondingly less precision, and imbalance of this degree raises the possibility of unmeasured differences that our baseline comparison lacked power to detect. Future trials in this setting should use blocked, site-stratified randomization with explicitly documented allocation concealment.

Fifth, structured health coaching was delivered to both arms. This trial therefore estimates the incremental value of wireless monitoring added to intensive coaching, not the value of wireless monitoring relative to usual care.

Sixth, adherence was measured by fundamentally different mechanisms in the 2 arms—automatic transmission versus voluntary return of a paper log—and the resulting figures are not comparable as measures of participant behavior. Nor were transmission failures logged separately from device nonuse in the wireless arm, so nonreceipt of a reading cannot be attributed to either cause.

Seventh, BP targets and their reporting were not fully aligned. Treatment during the study followed the age-stratified JNC8 thresholds current at the time and consistent with the HEDIS quality measures then in force, whereas BP control is reported here using a single systolic threshold of <140 mm Hg for all participants. We could not recompute control rates using the age-stratified definition because individual-level data are no longer available. Readers should also note that the 2017 American College of Cardiology and American Heart Association guideline subsequently redefined hypertension and lowered treatment targets to <130/80 mm Hg for most adults [22]; a substantial proportion of participants classified here as controlled would not meet contemporary targets, and these control rates are not directly comparable with those of trials conducted under current guidance.

Eighth, data were collected between 2015 and 2017 using a hub-and-cuff architecture (Qualcomm Life 2NET with a Bluetooth-enabled A&D cuff) that has since been superseded. Contemporary RPM programs typically rely on cellular-enabled cuffs or smartphone apps with direct electronic health record integration, automated alerting, and, in some cases, algorithmic titration support. Our findings are therefore best read as evidence about the human and organizational feasibility of remote BP monitoring in an FQHC population—whether patients of this profile will accept such a device, use it, and transmit data reliably—rather than as evidence about any currently marketed platform. We would expect the acceptability findings to transfer reasonably well because the low interaction burden of the system we studied resembles current cellular-enabled cuffs more closely than it resembles the smartphone app products that dominated the intervening period; the technical failure modes, in contrast, are likely to differ, and transmission reliability should be remeasured on any contemporary platform rather than assumed from these data.

Ninth, we collected no economic data. Device, hub, data plan, and staff time costs were absorbed within the parent HSF-Z program and were not tracked at the level of this substudy, so we cannot report cost per participant, cost per mm Hg of BP reduction, or any incremental cost-effectiveness estimate. As the case for RPM in safety-net settings often turns on cost-effectiveness and on whether reimbursement covers program costs, this is a material gap for policymakers and health center administrators, and prospective cost capture should be built into subsequent studies.

Finally, several more circumscribed limitations should be noted. The study was conducted in 3 FQHC networks within a single county, and findings may not generalize to other regions or to FQHC populations of different linguistic or demographic composition. Implementation metrics valuable for program planning—transmission failure counts, technical support contacts, device replacements, and setup assistance events—were not captured as discrete variables. The satisfaction instrument was investigator developed rather than psychometrically validated, was administered only to the wireless arm, and reached only participants still engaged at 6 months, so acceptability estimates are subject to responder bias. In addition, BP was ascertained by routine clinic measurement rather than by a blinded, standardized research protocol, which introduces measurement variability, although randomization should distribute that variability similarly across arms.

### Conclusions

This pilot shows that wireless HBPM can be implemented in an underserved, predominantly Hispanic FQHC population. Among analyzed wireless-arm participants, most transmitted data at high rates without any active reporting effort, and survey respondents reported little difficulty setting up or using the equipment and a preference for the wireless cuff over a conventional one. These estimates apply to participants who remained in the analyzed sample and, for acceptability, to survey respondents; feasibility among all randomized participants could not be estimated.

The broader implication concerns infrastructure rather than efficacy. A safety-net population often presumed to be a poor candidate for digital health—on grounds of income, language, or digital literacy—engaged at high rates with a connected monitoring device several years before the pandemic normalized remote care, at least among participants who remained in follow-up. That argues against excluding such populations from RPM programs on presumed capability grounds, and in favor of designing those programs around low-interaction devices together with a defined pathway for the minority of patients who do not engage. What this pilot cannot establish is whether wireless monitoring improves BP control beyond what intensive health coaching already achieves. Answering that question will require an adequately powered trial with intention-to-treat analysis, blocked and stratified randomization with concealed allocation, prospective cost capture, and a control condition that isolates the monitoring component; we hope the feasibility and acceptability estimates reported here are useful in designing it.

## Supplementary material

10.2196/100115 Multimedia Appendix 1 Operational workflows for the pilot. The upper panel traces the participant pathway from clinic-based identification and eligibility screening, through assignment to the wireless or standard arm, device receipt and first clinic blood pressure measurement, home monitoring, and escalation of out-of-range readings to the site registered nurse, health coach, and clinic physician for possible medication change. The lower panel shows alert routing across the 3 participating clinic networks, in which transmitted readings are aggregated centrally and high or low blood pressure alerts, feedback, and reminders are returned to site staff, health coaches, and patients.

10.2196/100115 Checklist 1 CONSORT-EHEALTH checklist (V 1.6.1).

## Abbreviations

AOR: adjusted odds ratio
BP: blood pressure
CONSORT-EHEALTH: Consolidated Standards of Reporting Trials of Electronic and Mobile Health Applications and Online Telehealth
FQHC: federally qualified health center
HBPM: home blood pressure monitoring
HEDIS: Healthcare Effectiveness Data and Information Set
HIPAA: Health Insurance Portability and Accountability Act
HSF-Z: Heart Attack and Stroke Free Zone
JNC8: Eighth Joint National Committee
MCAR: missing completely at random
RPM: remote patient monitoring
