# Supplementary material for: Wireless Home Blood Pressure Monitoring With Health Coaching for Hypertension Management at a Federally Qualified Health Center: Pilot Randomized Controlled Feasibility and Acceptability Study
Source: JMIR Form Res. 2026 Oct 22;10:e100115. doi: 10.2196/100115 (PMC13690290; doi:10.2196/100115)
Supplement: Supplementary file 2 [file formative-v10-e100115-s001.docx]

**Appendix A**

**2NET Deployment Proposed Flow:**

Clinic Site recognizes patient fitting proposed inclusion criteria

Flags patient to health coach vs protocol RN

Patient is protocoled to wireless vs non-wireless

Phone vs site encounter to discuss program; if pt meets criteria, is interested, 2NET system requirements screened

Patient receives non wireless BP monitor; Day 1 of study patient has clinic site BP

Patient receives 2NET system and installs system; Day 1 of study patient has clinic site BP measurement and then initiates home monitoring

Health coaches routinely monitor patients; >5 days not controlled referral to RN 🡪 MD or Pharmacist change

Patient continues monitoring twice daily x 1 month, then daily

Patient continues monitoring twice daily x 1 month, then daily

FLAG

Acute vs follow clinic vist

Clinical RN at site is notified who reviews log and contacts pt

Patient follows up in clinic routinely with home BP log, which is reviewed and possible medication change

High or Low measurements (>170/100) / (<100/50)

Patient follows up in clinic routinely; 2NET system reviewed; possible medication change

Clinic 1

N=?

Clinic 3

N=?

Clinic 2

N=?

C-TIP

High/low BP alerts

Clinic RN/tech

Patients/coaches

Feedback

Clinic MD

Reminder alerts
